# 4417 Association between Brain Volumes and Posttraumatic Stress Disorder in Intensive Care Unit Survivors

**DOI:** 10.1017/cts.2020.106

**Published:** 2020-07-29

**Authors:** Jo Ellen Wilson, Kristina Stepanovic, Baxter Rogers, Amy Kiehl, E. “Wes” Ely, James Jackson

**Affiliations:** 1 Vanderbilt University Medical Center

## Abstract

OBJECTIVES/GOALS: To explore the severity of posttraumatic stress disorder (PTSD) symptoms in association with hippocampal and amygdala volumes in ICU survivors. We hypothesize that the severity of posttraumatic stress symptoms in ICU survivors is associated with lower volumes of both the hippocampus and amygdala. METHODS/STUDY POPULATION: Secondary analysis of the VISIONS study, a prospective sub-study of the BRAIN-ICU cohort, which included survivors of critical illness. Patients were screened for preexisting PTSD before discharge. The PTSD Checklist Specific (PCL-S) was used at 3 and 12 months to evaluate the ICU as a traumatic experience. A score of >30, indicated significant symptoms of PTSD. A Philips Achieva 3T MRI scanner was used to scan patients at both discharge and 3-month follow-up. To compare median brain volumes at discharge and 3 months for those with and without significant PTSD symptomatology (PCL-S ≥30) at 3 and 12 months, we used a Kruskal-Wallis (KW) equality-of-populations rank test. RESULTS/ANTICIPATED RESULTS: The median age for our sample was 58.5 (52.6, 63.7). One-third of the sample was female, and 90% were Caucasian. Fifty-seven percent of individuals (N = 12) had at least one prior mental health diagnosis, with two having a prior history of PTSD. One third of individuals experienced delirium during their critical illness. At 3-month follow up, there were three patients with PTSD symptomatology and one at 12-month follow up. Median brain volumes (hippocampus or amygdala) did not differ between individuals with or without PTSD symptomatology at either 3 or 12 months (p-values for all tests >0.05). DISCUSSION/SIGNIFICANCE OF IMPACT: Although our study did not reveal significant differences in brain volumes between PTSD patients and non-PTSD patients, sample size is a major limitation and larger scale studies should be undertaken to elucidate possible neurobiological markers of PTSD in ICU survivors. CONFLICT OF INTEREST DESCRIPTION: Dr. Wilson would like to acknowledge salary support from the Vanderbilt Faculty Research Scholars Program (1KL2TR002245), HL111111 and GM120484. Drs. Ely and Jackson as well as Mrs. Kiehl all receive funding for their time working on this investigation from AG035117 and HL111111. Dr. Ely would additionally like to acknowledge salary support from the Tennessee Valley Healthcare System Geriatric Research Education and Clinical Center (GRECC). Dr. Ely will also disclose additional funding for his time from AG027472 and having received honoraria from Orion and Hospira for CME activity; he does not hold stock or consultant relationships with those companies. The authors would like to acknowledge the following: this work was conducted in part using the resources of the Center for Computational Imaging at Vanderbilt University Institute of Imaging Science and the Advanced Computing Center for Research and Education at Vanderbilt University, Nashville, TN, and study data were collected and managed using REDCap electronic data capture tools hosted at Vanderbilt University.

